# Na_3.88_Mo_15_Se_19_: a novel ternary reduced molybdenum selenide containing Mo_6_ and Mo_9_ clusters

**DOI:** 10.1107/S1600536813025907

**Published:** 2013-09-25

**Authors:** Diala Salloum, Patrick Gougeon, Philippe Gall

**Affiliations:** a Faculty of Science III, Lebanese University, PO Box 826, Kobbeh-Tripoli, Lebanon; b Unité Sciences Chimiques de Rennes, UMR CNRS No. 6226, Université de Rennes I - INSA Rennes, Campus de Beaulieu, 35042 Rennes CEDEX, France

## Abstract

The structure of tetrasodium penta­deca­molybdenum nona­deca­selenide, Na_3.88_Mo_15_Se_19_, is isotypic with the In_3+*x*_Mo_15_Se_19_ compounds [Grüttner *et al.* (1979 ▶). *Acta Cryst.* B**35**, 285–292]. It is characterized by two cluster units, Mo_6_Se^*i*^_8_Se^*a*^_6_ and Mo_9_Se^*i*^_11_Se^*a*^_6_ (where *i* represents inner and *a* apical atoms), that are present in a 1:1 ratio. The cluster units are centered at Wyckoff positions 2*b* and 2*c* and have point-group symmetry -3 and -6, respectively. The clusters are inter­connected through additional Mo—Se bonds. In the title compound, the Na^+^ cations replace the trivalent as well as the monovalent indium atoms present in In_3.9_Mo_15_Se_19_. One Mo, one Se and one Na atom are situated on mirror planes, and two other Se atoms and one Na atom [occupancy 0.628 (14)] are situated on threefold rotation axes. The crystal studied was twinned by merohedry with refined components of 0.4216 (12) and 0.5784 (12).

## Related literature

For previous reports on the crystal structures of In_3_Mo_15_Se_19_ compounds, see: Grüttner *et al.* (1979 ▶). For physical properties of this type of compounds, see: Seeber *et al.* (1979 ▶). The crystal structures of the substituted selenides Ho_0.76_In_1.68_Mo_15_Se_19_ and In_0.87_K_2_Mo_15_Se_19_ were reported by Salloum *et al.* (2006 ▶, 2007 ▶). For the isotypic sulfides In_3.7_Mo_15_S_19_, In_1.6_Rb_2_Mo_15_S_19_, In_2.2_CsMo_15_S_19_ and ScTl_2_Mo_15_S_19_, see: Salloum *et al.* (2004*a* ▶,*b* ▶); Gougeon *et al.* (2010 ▶). For details of the *i*- and *a*-type ligand notation, see: Schäfer & von Schnering (1964 ▶).

## Experimental

### 

#### Crystal data

Na_3.88_Mo_15_Se_19_
*M* *_r_* = 3028.54
Hexagonal, 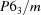

*a* = 9.8647 (1) Å
*c* = 19.5957 (3) Å
*V* = 1651.43 (3) Å^3^
*Z* = 2
Mo *K*α radiation
μ = 26.47 mm^−1^
*T* = 293 K
0.09 × 0.07 × 0.06 mm

#### Data collection

Nonius KappaCCD diffractometer
Absorption correction: analytical (de Meulenaer & Tompa, 1965 ▶) *T*_min_ = 0.111, *T*_max_ = 0.215
25999 measured reflections
2487 independent reflections
2116 reflections with *I* > 2σ(*I*)
*R*_int_ = 0.075

#### Refinement

*R*[*F* ^2^ > 2σ(*F* ^2^)] = 0.033
*wR*(*F* ^2^) = 0.084
*S* = 1.06
2487 reflections
67 parameters
Δρ_max_ = 2.69 e Å^−3^
Δρ_min_ = −1.81 e Å^−3^

### 

Data collection: *COLLECT* (Nonius, 1998 ▶); cell refinement: *COLLECT*; data reduction: *EVALCCD* (Duisenberg, 1998 ▶); program(s) used to solve structure: *SIR97* (Altomare *et al.*, 1999 ▶); program(s) used to refine structure: *SHELXL97* (Sheldrick, 2008 ▶); molecular graphics: *DIAMOND* (Bergerhoff, 1996 ▶); software used to prepare material for publication: *SHELXL97* and *PLATON* (Spek, 2009 ▶).

## Supplementary Material

Crystal structure: contains datablock(s) I, global. DOI: 10.1107/S1600536813025907/ru2054sup1.cif

Structure factors: contains datablock(s) I. DOI: 10.1107/S1600536813025907/ru2054Isup2.hkl

Additional supplementary materials:  crystallographic information; 3D view; checkCIF report

## Figures and Tables

**Table 1:** Selected bond lengths (Å)

Mo1—Se4^i^	2.5287 (7)
Mo1—Se1^ii^	2.5600 (6)
Mo1—Se1	2.5810 (7)
Mo1—Se1^iii^	2.6075 (7)
Mo1—Mo1^iii^	2.6980 (7)
Mo1—Mo1^ii^	2.7152 (7)
Mo1—Se2^iv^	2.7207 (7)
Mo2—Se5	2.5220 (7)
Mo2—Se2	2.6023 (8)
Mo2—Mo2^v^	2.6311 (7)
Mo2—Se2^vi^	2.6312 (8)
Mo2—Se3	2.6830 (6)
Mo2—Se1	2.7018 (7)
Mo2—Mo3^vi^	2.7153 (6)
Mo2—Mo3	2.7537 (6)
Mo3—Se3	2.5586 (11)
Mo3—Se3^v^	2.5675 (11)
Mo3—Se2	2.6153 (6)
Mo3—Se2^vii^	2.6153 (6)
Mo3—Mo3^vi^	2.6834 (11)
Se5—Na1	2.848 (6)
Na1—Se2^viii^	3.2010 (11)
Na1—Se1^ix^	3.462 (2)
Na2—Se3^v^	2.657 (5)
Na2—Se4^vii^	2.747 (4)
Na2—Se2^x^	2.812 (4)
Na2—Se3^xi^	3.096 (5)

Symmetry codes: (i) 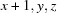; (ii) 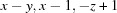; (iii) 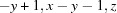; (iv) 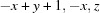; (v) 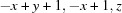; (vi) 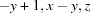; (vii) 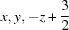; (viii) 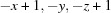; (ix) 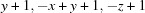; (x) 
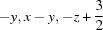
; (xi) 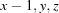.

**Fig. 1.**
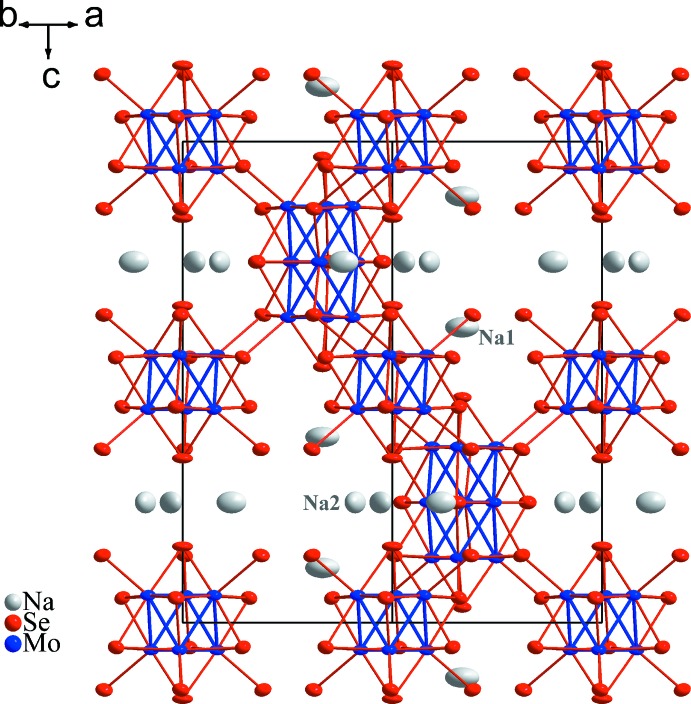
: View of Na3.9Mo15Se19 along [110]. Displacement ellipsoids are drawn at the 97% probability level.

**Fig. 2.**
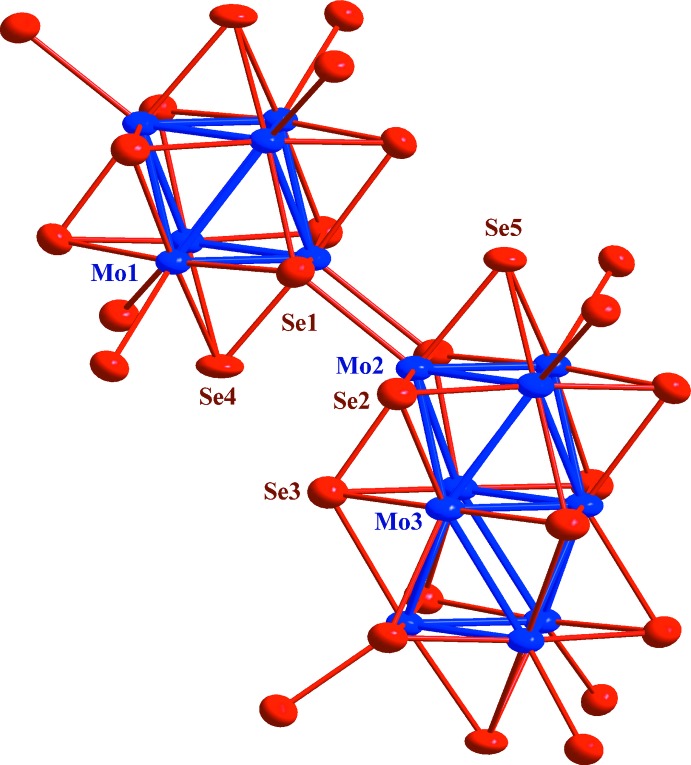
Plot showing the atom-numbering scheme and the interunit linkage of the Mo9Se11Se6 and Mo6Se8Se6 cluster units. Displacement ellipsoids are drawn at the 97% probability level.

## supplementary crystallographic information

### 1. Comment

The reduced molybdenum compounds In_3 +_*_x_*Mo_15_*X*_19_ (*X* = S, Se) (Grüttner *et al.*, 1979; Salloum *et al.*, 2004*a*) crystallize in an interesting structural type characterized by an equal mixture of Mo_6_ and Mo_9_ clusters and by In atoms that occupy two or three different crystallographically positions depending on their formal oxidation state of +1 or +3. Subsequently, isomorphous compounds such as Ho_0.76_In_1.68_Mo_15_Se_19_ (Salloum *et al.*, 2006), In_0.87_K_2_Mo_15_Se_19_ (Salloum *et al.*, 2007), V_1.42_In_1.83_Mo_15_Se_19_ (Gougeon *et al.*, 2010), In_3.7_Mo_15_S_19_ (Salloum *et al.*, 2004*a*), In_1.6_Rb_2_Mo_15_S_19_, In_2.2_CsMo_15_S_19_ and ScTl_2_Mo_15_S_19_ (Salloum *et al.*, 2004*b*) have been synthesized. In the latter compounds, the Ho, V and Sc atoms replace the trivalent indium and the K, Cs, and Tl atoms the monovalent one. We present here the crystal structure of Na_3.9_Mo_15_Se_19_ in which the sodium replaces the monovalent as well as the trivalent indium for the first time.

The Mo—Se framework of the title compound consists of the cluster units Mo_6_Se^i^_8_Se*^a^*_6_ and Mo_9_Se^i^_11_Se*^a^*_6_ in a 1:1 ratio (for details of the i- and a-type ligand notation, see Schäfer & von Schnering (1964)). Both components are interconnected through additional Mo—Se bonds (Figs. 1 and 2). The first unit can be described as an Mo_6_ octahedron surrounded by eight face-capping inner Se^i^ and six apical Se*^a^* ligands. The Mo_9_ cluster is surrounded by 11 Se^i^ atoms capping one or two faces of the bioctahedron and six Se*^a^* ligands above the apical Mo atoms. The Mo_6_Se^i^_8_Se*^a^*_6_ and Mo_9_Se^i^_11_Se*^a^*_6_ units are centered at Wyckoff positions 2 b and 2c and have point-group symmetry 3 and 6, respectively. The Mo—Mo distances within the Mo_6_ cluster are 2.6980 (7) Å for the distances of the Mo triangles formed by the Mo1 atoms related through the threefold axis, and 2.7152 (7) Å for the distances between these triangles. The Mo—Mo distances within the Mo_9_ clusters are 2.6311 (7) and 2.6834 (11) Å in the triangles formed by the atoms Mo2 and Mo3, respectively, and 2.7153 (6) and 2.7537 (6) Å for those between the Mo2_3_ and Mo3_3_ triangles. The Se atoms bridge either one (Se1, Se2, Se4 and Se5) or two (Se3) triangular faces of the Mo clusters. Moreover, atoms Se1 and Se2 are linked to an Mo atom of a neighboring cluster. The Mo—Se bond distances range from 2.5287 (7) to 2.7207 (7) Å within the Mo_6_Se^i^_8_Se*^a^*_6_ unit, and from 2.5220 (7) to 2.7018 (7) Å within the Mo_9_Se^i^_11_Se*^a^*_6_ unit. In both cases, the shortest bonds involve the Se4 and Se5 terminal atoms and the longest ones correspond to the interunit Mo1—Se2 and Mo2—Se1 bonds. Each Mo_9_Se^i^_11_Se*^a^*_6_ cluster is thus interconnected to six Mo_6_Se^i^_8_Se*^a^*_6_ units (and *vice versa*) *via* Mo2—Se1 bonds (and Mo1—Se2 bonds, respectively), forming the three-dimensional Mo—Se framework, the connective formula of which is Mo_9_Se^i^_5_Se^i-a^_6/2_Se^a-i^_6/2_, Mo_6_Se^i^_2_Se^i-a^_6/2_Se^a-i^_6/2_. It results from this arrangement that the shortest intercluster Mo1—Mo2 distance is 3.5202 (6) Å, indicating only weak metal-metal interactions between the Mo clusters. The Na^+^ cations are surrounded by seven Se atoms forming a distorted tricapped tetrahedron, as is the case in In_3 +_*_x_*Mo_15_Se_19_. The Se5 and Se2 atoms forming the tetrahedron are at 2.848 (6) and 3.2010 (11) Å from the Na1 atom, and the capping Se1 atoms are at 3.462 (2) Å. The Na2^+^ cations, as the In^3+^ cations in the In_3.9_Mo_15_Se_19_ compounds, occupy partially at 62.7% a triangular group of distorted octahedral cavities around the threefold axis, which are formed by two Mo_6_Se^i^_8_Se*^a^*_6_ and three Mo_9_Se^i^_11_Se*^a^*_6_ units. The Na2—Se distances are in the 2.657 (5) - 3.096 (5) Å range.

### 2. Experimental

Single crystals of Na_3.9_Mo_15_Se_19_ were prepared from an ion exchange reaction on single crystals of In_3 +_*_x_*Mo_15_Se_19_ with an excess of NaI at 1073 K. The mixture was sealed under vacuum in a long silica tube. The end of tube containing the crystals of In_3 +_*_x_*Mo_15_Se_19_ and InI was placed in a furnace with about 5 cm of the other end out from the furnace, at about the room temperature. The furnace was heated at 1073 K for 48 h. After reaction, crystals of InI were observed at the cool end of the tube. The black crystals of the title compound were subsequently washed with water to remove the excess of InI. Qualitative microanalyses using a Jeol JSM 6400 scanning electron microscope equipped with a Oxford INCA energy- dispersive-type X-ray spectrometer did not reveal the presence of indium in the crystals and indicated roughly stoichiometries comprised between 3.6 and 4.2 for the Na content.

### 3. Refinement

Analysis of the intensity data using the TwinRotMat routine of *PLATON* (Spek, 2009) revealed the studied crystal was twinned by merohedry with [110, 010, 001] as the twin matrix. The ratio of the twin components was refined to 0.4216 (12):0.5784 (12). No significant deviation from full occupancy was observed for Na1. The site occupation factor of Na2 was refined freely leading to the final stoichiometry Na_3.88 (4)_Mo_15_Se_19_.

### Crystal data

**Table:**

Na_3.88_Mo_15_Se_19_	*D*_x_ = 6.091 Mg m^−^^3^
*M**_r_* = 3028.54	Mo *K*α radiation, λ = 0.71069 Å
Hexagonal, *P*6_3_/*m*	Cell parameters from 26001 reflections
*a* = 9.8647 (1) Å	θ = 1.7–35.0°
*c* = 19.5957 (3) Å	µ = 26.47 mm^−^^1^
*V* = 1651.43 (3) Å^3^	*T* = 293 K
*Z* = 2	Multi-faceted crystal, black
*F*(000) = 2637	0.09 × 0.07 × 0.06 mm

### Data collection

**Table:**

Nonius KappaCCD diffractometer	2487 independent reflections
Radiation source: fine-focus sealed tube	2116 reflections with *I* > 2σ(*I*)
Graphite monochromator	*R*_int_ = 0.075
φ scans (κ = 0) + additional ω scans	θ_max_ = 35.0°, θ_min_ = 2.4°
Absorption correction: analytical (de Meulenaer & Tompa, 1965)	*h* = −15→15
*T*_min_ = 0.111, *T*_max_ = 0.215	*k* = −15→15
25999 measured reflections	*l* = −19→31

### Refinement

**Table:**

Refinement on *F*^2^	Primary atom site location: structure-invariant direct methods
Least-squares matrix: full	Secondary atom site location: difference Fourier map
*R*[*F*^2^ > 2σ(*F*^2^)] = 0.033	*w* = 1/[σ^2^(*F*_o_^2^) + (0.0435*P*)^2^ + 3.7236*P*] where *P* = (*F*_o_^2^ + 2*F*_c_^2^)/3
*wR*(*F*^2^) = 0.084	(Δ/σ)_max_ = 0.001
*S* = 1.06	Δρ_max_ = 2.69 e Å^−^^3^
2487 reflections	Δρ_min_ = −1.81 e Å^−^^3^
67 parameters	Extinction correction: *SHELXL*, Fc^*^=kFc[1+0.001xFc^2^λ^3^/sin(2θ)]^-1/4^
0 restraints	Extinction coefficient: 0.00058 (8)

### Special details

**Table:**

Geometry. All e.s.d.'s (except the e.s.d. in the dihedral angle between two l.s. planes) are estimated using the full covariance matrix. The cell e.s.d.'s are taken into account individually in the estimation of e.s.d.'s in distances, angles and torsion angles; correlations between e.s.d.'s in cell parameters are only used when they are defined by crystal symmetry. An approximate (isotropic) treatment of cell e.s.d.'s is used for estimating e.s.d.'s involving l.s. planes.
Refinement. Refinement of *F*^2^ against ALL reflections. The weighted *R*-factor *wR* and goodness of fit *S* are based on *F*^2^, conventional *R*-factors *R* are based on *F*, with *F* set to zero for negative *F*^2^. The threshold expression of *F*^2^ > σ(*F*^2^) is used only for calculating *R*-factors(gt) *etc*. and is not relevant to the choice of reflections for refinement. *R*-factors based on *F*^2^ are statistically about twice as large as those based on *F*, and *R*- factors based on ALL data will be even larger.

### Fractional atomic coordinates and isotropic or equivalent isotropic displacement parameters (Å^2^)

**Table:**

	*x*	*y*	*z*	*U*_iso_*/*U*_eq_	Occ. (<1)
Mo1	0.84973 (5)	−0.16457 (5)	0.556745 (19)	0.01378 (9)	
Mo2	0.68549 (6)	0.18962 (5)	0.63450 (2)	0.01510 (9)	
Mo3	0.51739 (7)	0.16952 (8)	0.7500	0.01574 (11)	
Se1	0.68259 (6)	−0.03045 (6)	0.55145 (2)	0.01653 (11)	
Se2	0.38303 (7)	0.00893 (7)	0.63968 (2)	0.01760 (11)	
Se3	0.69343 (10)	0.04771 (10)	0.7500	0.01993 (16)	
Se4	0.0000	0.0000	0.65840 (4)	0.0222 (2)	
Se5	0.6667	0.3333	0.53176 (4)	0.01851 (18)	
Na1	0.6667	0.3333	0.3864 (3)	0.0618 (18)	
Na2	0.0558 (6)	0.2330 (7)	0.7500	0.0214 (16)	0.628 (14)

### Atomic displacement parameters (Å^2^)

**Table:**

	*U*^11^	*U*^22^	*U*^33^	*U*^12^	*U*^13^	*U*^23^
Mo1	0.01667 (19)	0.01711 (19)	0.00794 (16)	0.00872 (17)	0.00032 (12)	0.00005 (13)
Mo2	0.0186 (2)	0.0185 (2)	0.00811 (15)	0.00916 (17)	−0.00004 (14)	−0.00039 (13)
Mo3	0.0195 (3)	0.0198 (3)	0.0079 (2)	0.0097 (2)	0.000	0.000
Se1	0.0181 (2)	0.0198 (3)	0.01279 (19)	0.0103 (2)	0.00148 (17)	−0.00079 (17)
Se2	0.0198 (2)	0.0200 (2)	0.01247 (19)	0.0095 (2)	−0.00268 (17)	−0.00173 (17)
Se3	0.0252 (4)	0.0232 (4)	0.0131 (3)	0.0134 (3)	0.000	0.000
Se4	0.0297 (3)	0.0297 (3)	0.0071 (3)	0.01486 (15)	0.000	0.000
Se5	0.0239 (3)	0.0239 (3)	0.0078 (3)	0.01194 (14)	0.000	0.000
Na1	0.079 (3)	0.079 (3)	0.028 (3)	0.0393 (15)	0.000	0.000
Na2	0.017 (3)	0.035 (3)	0.019 (2)	0.018 (2)	0.000	0.000

### Geometric parameters (Å, º)

**Table:**

Mo1—Se4^i^	2.5287 (7)	Se1—Na1^xiii^	3.462 (2)
Mo1—Se1^ii^	2.5600 (6)	Se2—Mo2^ix^	2.6312 (8)
Mo1—Se1	2.5810 (7)	Se2—Mo1^xiv^	2.7207 (7)
Mo1—Se1^iii^	2.6075 (7)	Se2—Na2^xii^	2.812 (4)
Mo1—Mo1^iii^	2.6980 (7)	Se2—Na1^xiii^	3.2010 (11)
Mo1—Mo1^iv^	2.6980 (7)	Se3—Mo3^viii^	2.5675 (11)
Mo1—Mo1^ii^	2.7152 (7)	Se3—Na2^viii^	2.657 (5)
Mo1—Mo1^v^	2.7152 (7)	Se3—Mo2^x^	2.6830 (6)
Mo1—Se2^vi^	2.7207 (7)	Se3—Na2^i^	3.096 (5)
Mo1—Mo2^iii^	3.5202 (6)	Se4—Mo1^xv^	2.5287 (7)
Mo1—Mo1^vii^	3.8278 (8)	Se4—Mo1^xiv^	2.5287 (7)
Mo1—Na2^viii^	3.8628 (12)	Se4—Mo1^ix^	2.5287 (7)
Mo2—Se5	2.5220 (7)	Se4—Na2^xii^	2.747 (4)
Mo2—Se2	2.6023 (8)	Se4—Na2	2.747 (4)
Mo2—Mo2^ix^	2.6311 (7)	Se4—Na2^xvi^	2.747 (5)
Mo2—Mo2^viii^	2.6311 (7)	Se5—Mo2^viii^	2.5220 (7)
Mo2—Se2^viii^	2.6312 (8)	Se5—Mo2^ix^	2.5220 (7)
Mo2—Se3	2.6830 (6)	Se5—Na1	2.848 (6)
Mo2—Se1	2.7018 (7)	Na1—Se2^xiii^	3.2010 (11)
Mo2—Mo3^viii^	2.7153 (6)	Na1—Se2^v^	3.2010 (11)
Mo2—Mo3	2.7537 (6)	Na1—Se2^xvii^	3.2010 (11)
Mo2—Mo1^iv^	3.5202 (6)	Na1—Se1^v^	3.462 (2)
Mo3—Se3	2.5586 (11)	Na1—Se1^xiii^	3.462 (2)
Mo3—Se3^ix^	2.5675 (11)	Na1—Se1^xvii^	3.462 (2)
Mo3—Se2	2.6153 (6)	Na2—Se3^ix^	2.657 (5)
Mo3—Se2^x^	2.6153 (6)	Na2—Se4^x^	2.747 (4)
Mo3—Mo3^viii^	2.6834 (11)	Na2—Se2^xviii^	2.812 (4)
Mo3—Mo3^ix^	2.6834 (11)	Na2—Se2^xvi^	2.812 (4)
Mo3—Mo2^xi^	2.7153 (6)	Na2—Mo3^xvi^	2.956 (5)
Mo3—Mo2^ix^	2.7153 (6)	Na2—Se3^xv^	3.096 (5)
Mo3—Mo2^x^	2.7537 (6)	Na2—Na2^xvi^	3.601 (10)
Mo3—Na2^xii^	2.956 (5)	Na2—Na2^xii^	3.601 (10)
Se1—Mo1^v^	2.5600 (6)	Na2—Mo1^xi^	3.8628 (12)
Se1—Mo1^iv^	2.6075 (7)	Na2—Mo1^ix^	3.8628 (12)
			
Se4^i^—Mo1—Se1^ii^	176.02 (2)	Mo3^ix^—Mo3—Mo2	89.039 (15)
Se4^i^—Mo1—Se1	91.274 (18)	Mo2^xi^—Mo3—Mo2	146.42 (3)
Se1^ii^—Mo1—Se1	89.153 (17)	Mo2^ix^—Mo3—Mo2	57.508 (17)
Se4^i^—Mo1—Se1^iii^	90.664 (18)	Mo2^x^—Mo3—Mo2	110.55 (3)
Se1^ii^—Mo1—Se1^iii^	88.572 (18)	Se3—Mo3—Na2^xii^	115.38 (12)
Se1—Mo1—Se1^iii^	174.67 (3)	Se3^ix^—Mo3—Na2^xii^	67.75 (12)
Se4^i^—Mo1—Mo1^iii^	57.759 (12)	Se2—Mo3—Na2^xii^	60.24 (4)
Se1^ii^—Mo1—Mo1^iii^	118.706 (18)	Se2^x^—Mo3—Na2^xii^	60.24 (4)
Se1—Mo1—Mo1^iii^	119.10 (2)	Mo3^viii^—Mo3—Na2^xii^	173.98 (12)
Se1^iii^—Mo1—Mo1^iii^	58.19 (2)	Mo3^ix^—Mo3—Na2^xii^	126.02 (12)
Se4^i^—Mo1—Mo1^iv^	57.759 (12)	Mo2^xi^—Mo3—Na2^xii^	93.47 (6)
Se1^ii^—Mo1—Mo1^iv^	119.357 (19)	Mo2^ix^—Mo3—Na2^xii^	93.47 (6)
Se1—Mo1—Mo1^iv^	59.15 (2)	Mo2^x^—Mo3—Na2^xii^	118.06 (4)
Se1^iii^—Mo1—Mo1^iv^	118.14 (2)	Mo2—Mo3—Na2^xii^	118.06 (4)
Mo1^iii^—Mo1—Mo1^iv^	60.0	Mo1^v^—Se1—Mo1	63.76 (2)
Se4^i^—Mo1—Mo1^ii^	117.937 (16)	Mo1^v^—Se1—Mo1^iv^	63.39 (2)
Se1^ii^—Mo1—Mo1^ii^	58.50 (2)	Mo1—Se1—Mo1^iv^	62.66 (2)
Se1—Mo1—Mo1^ii^	117.326 (19)	Mo1^v^—Se1—Mo2	132.81 (3)
Se1^iii^—Mo1—Mo1^ii^	57.453 (14)	Mo1—Se1—Mo2	129.41 (2)
Mo1^iii^—Mo1—Mo1^ii^	60.209 (10)	Mo1^iv^—Se1—Mo2	83.04 (2)
Mo1^iv^—Mo1—Mo1^ii^	90.0	Mo1^v^—Se1—Na1^xiii^	130.23 (8)
Se4^i^—Mo1—Mo1^v^	117.937 (16)	Mo1—Se1—Na1^xiii^	99.13 (2)
Se1^ii^—Mo1—Mo1^v^	59.16 (2)	Mo1^iv^—Se1—Na1^xiii^	151.91 (7)
Se1—Mo1—Mo1^v^	57.743 (15)	Mo2—Se1—Na1^xiii^	95.04 (6)
Se1^iii^—Mo1—Mo1^v^	117.026 (19)	Mo2—Se2—Mo3	63.71 (2)
Mo1^iii^—Mo1—Mo1^v^	90.0	Mo2—Se2—Mo2^ix^	60.36 (2)
Mo1^iv^—Mo1—Mo1^v^	60.209 (9)	Mo3—Se2—Mo2^ix^	62.333 (19)
Mo1^ii^—Mo1—Mo1^v^	59.583 (19)	Mo2—Se2—Mo1^xiv^	127.69 (2)
Se4^i^—Mo1—Se2^vi^	91.21 (2)	Mo3—Se2—Mo1^xiv^	130.42 (3)
Se1^ii^—Mo1—Se2^vi^	92.77 (2)	Mo2^ix^—Se2—Mo1^xiv^	82.24 (2)
Se1—Mo1—Se2^vi^	87.51 (2)	Mo2—Se2—Na2^xii^	129.48 (9)
Se1^iii^—Mo1—Se2^vi^	97.41 (2)	Mo3—Se2—Na2^xii^	65.90 (8)
Mo1^iii^—Mo1—Se2^vi^	136.97 (2)	Mo2^ix^—Se2—Na2^xii^	98.77 (11)
Mo1^iv^—Mo1—Se2^vi^	130.63 (2)	Mo1^xiv^—Se2—Na2^xii^	88.56 (10)
Mo1^ii^—Mo1—Se2^vi^	139.27 (2)	Mo2—Se2—Na1^xiii^	103.57 (2)
Mo1^v^—Mo1—Se2^vi^	132.73 (2)	Mo3—Se2—Na1^xiii^	122.35 (10)
Se4^i^—Mo1—Mo2^iii^	91.163 (16)	Mo2^ix^—Se2—Na1^xiii^	160.83 (7)
Se1^ii^—Mo1—Mo2^iii^	91.320 (19)	Mo1^xiv^—Se2—Na1^xiii^	102.71 (7)
Se1—Mo1—Mo2^iii^	135.27 (2)	Na2^xii^—Se2—Na1^xiii^	99.85 (14)
Se1^iii^—Mo1—Mo2^iii^	49.629 (15)	Mo3—Se3—Mo3^viii^	63.13 (3)
Mo1^iii^—Mo1—Mo2^iii^	99.53 (2)	Mo3—Se3—Na2^viii^	157.67 (13)
Mo1^iv^—Mo1—Mo2^iii^	148.233 (15)	Mo3^viii^—Se3—Na2^viii^	139.20 (13)
Mo1^ii^—Mo1—Mo2^iii^	100.519 (16)	Mo3—Se3—Mo2^x^	63.33 (2)
Mo1^v^—Mo1—Mo2^iii^	149.644 (19)	Mo3^viii^—Se3—Mo2^x^	62.236 (19)
Se2^vi^—Mo1—Mo2^iii^	47.784 (15)	Na2^viii^—Se3—Mo2^x^	121.81 (2)
Se4^i^—Mo1—Mo1^vii^	87.495 (19)	Mo3—Se3—Mo2	63.33 (2)
Se1^ii^—Mo1—Mo1^vii^	88.562 (18)	Mo3^viii^—Se3—Mo2	62.236 (19)
Se1—Mo1—Mo1^vii^	87.95 (2)	Na2^viii^—Se3—Mo2	121.81 (2)
Se1^iii^—Mo1—Mo1^vii^	87.18 (2)	Mo2^x^—Se3—Mo2	115.04 (3)
Mo1^iii^—Mo1—Mo1^vii^	45.181 (8)	Mo3—Se3—Na2^i^	125.24 (11)
Mo1^iv^—Mo1—Mo1^vii^	45.181 (8)	Mo3^viii^—Se3—Na2^i^	62.11 (11)
Mo1^ii^—Mo1—Mo1^vii^	44.819 (8)	Na2^viii^—Se3—Na2^i^	77.1 (2)
Mo1^v^—Mo1—Mo1^vii^	44.819 (8)	Mo2^x^—Se3—Na2^i^	91.04 (6)
Se2^vi^—Mo1—Mo1^vii^	175.25 (3)	Mo2—Se3—Na2^i^	91.04 (6)
Mo2^iii^—Mo1—Mo1^vii^	136.78 (2)	Mo1^xv^—Se4—Mo1^xiv^	64.48 (2)
Se4^i^—Mo1—Na2^viii^	45.18 (9)	Mo1^xv^—Se4—Mo1^ix^	64.48 (2)
Se1^ii^—Mo1—Na2^viii^	138.79 (9)	Mo1^xiv^—Se4—Mo1^ix^	64.48 (2)
Se1—Mo1—Na2^viii^	82.95 (8)	Mo1^xv^—Se4—Na2^xii^	127.86 (10)
Se1^iii^—Mo1—Na2^viii^	101.91 (8)	Mo1^xiv^—Se4—Na2^xii^	94.05 (8)
Mo1^iii^—Mo1—Na2^viii^	100.18 (9)	Mo1^ix^—Se4—Na2^xii^	149.31 (10)
Mo1^iv^—Mo1—Na2^viii^	90.73 (8)	Mo1^xv^—Se4—Na2	149.31 (9)
Mo1^ii^—Mo1—Na2^viii^	156.33 (8)	Mo1^xiv^—Se4—Na2	127.86 (10)
Mo1^v^—Mo1—Na2^viii^	138.84 (7)	Mo1^ix^—Se4—Na2	94.05 (8)
Se2^vi^—Mo1—Na2^viii^	46.69 (8)	Na2^xii^—Se4—Na2	81.92 (12)
Mo2^iii^—Mo1—Na2^viii^	67.91 (7)	Mo1^xv^—Se4—Na2^xvi^	94.05 (8)
Mo1^vii^—Mo1—Na2^viii^	131.19 (9)	Mo1^xiv^—Se4—Na2^xvi^	149.31 (9)
Se5—Mo2—Se2	91.991 (19)	Mo1^ix^—Se4—Na2^xvi^	127.86 (10)
Se5—Mo2—Mo2^ix^	58.558 (12)	Na2^xii^—Se4—Na2^xvi^	81.92 (12)
Se2—Mo2—Mo2^ix^	60.37 (3)	Na2—Se4—Na2^xvi^	81.92 (12)
Se5—Mo2—Mo2^viii^	58.558 (12)	Mo2^viii^—Se5—Mo2	62.88 (2)
Se2—Mo2—Mo2^viii^	120.31 (3)	Mo2^viii^—Se5—Mo2^ix^	62.88 (2)
Mo2^ix^—Mo2—Mo2^viii^	60.0	Mo2—Se5—Mo2^ix^	62.88 (2)
Se5—Mo2—Se2^viii^	91.314 (18)	Mo2^viii^—Se5—Na1	142.963 (15)
Se2—Mo2—Se2^viii^	175.53 (3)	Mo2—Se5—Na1	142.963 (15)
Mo2^ix^—Mo2—Se2^viii^	119.22 (2)	Mo2^ix^—Se5—Na1	142.963 (15)
Mo2^viii^—Mo2—Se2^viii^	59.27 (2)	Se5—Na1—Se2^xiii^	99.20 (10)
Se5—Mo2—Se3	175.05 (3)	Se5—Na1—Se2^v^	99.20 (10)
Se2—Mo2—Se3	86.12 (2)	Se2^xiii^—Na1—Se2^v^	117.49 (6)
Mo2^ix^—Mo2—Se3	116.64 (2)	Se5—Na1—Se2^xvii^	99.20 (10)
Mo2^viii^—Mo2—Se3	118.69 (2)	Se2^xiii^—Na1—Se2^xvii^	117.49 (6)
Se2^viii^—Mo2—Se3	90.35 (3)	Se2^v^—Na1—Se2^xvii^	117.49 (6)
Se5—Mo2—Se1	89.81 (2)	Se5—Na1—Se1^v^	69.42 (9)
Se2—Mo2—Se1	85.71 (2)	Se2^xiii^—Na1—Se1^v^	66.67 (2)
Mo2^ix^—Mo2—Se1	129.77 (2)	Se2^v^—Na1—Se1^v^	65.44 (2)
Mo2^viii^—Mo2—Se1	137.17 (2)	Se2^xvii^—Na1—Se1^v^	168.60 (19)
Se2^viii^—Mo2—Se1	97.31 (2)	Se5—Na1—Se1^xiii^	69.42 (9)
Se3—Mo2—Se1	94.60 (2)	Se2^xiii^—Na1—Se1^xiii^	65.44 (2)
Se5—Mo2—Mo3^viii^	120.52 (2)	Se2^v^—Na1—Se1^xiii^	168.60 (19)
Se2—Mo2—Mo3^viii^	117.08 (2)	Se2^xvii^—Na1—Se1^xiii^	66.67 (2)
Mo2^ix^—Mo2—Mo3^viii^	90.964 (15)	Se1^v^—Na1—Se1^xiii^	108.34 (9)
Mo2^viii^—Mo2—Mo3^viii^	61.980 (17)	Se5—Na1—Se1^xvii^	69.42 (9)
Se2^viii^—Mo2—Mo3^viii^	58.547 (18)	Se2^xiii^—Na1—Se1^xvii^	168.60 (19)
Se3—Mo2—Mo3^viii^	56.80 (2)	Se2^v^—Na1—Se1^xvii^	66.67 (2)
Se1—Mo2—Mo3^viii^	139.02 (2)	Se2^xvii^—Na1—Se1^xvii^	65.44 (2)
Se5—Mo2—Mo3	119.05 (2)	Se1^v^—Na1—Se1^xvii^	108.34 (9)
Se2—Mo2—Mo3	58.376 (18)	Se1^xiii^—Na1—Se1^xvii^	108.34 (9)
Mo2^ix^—Mo2—Mo3	60.511 (18)	Se3^ix^—Na2—Se4	87.67 (13)
Mo2^viii^—Mo2—Mo3	90.119 (16)	Se3^ix^—Na2—Se4^x^	87.67 (13)
Se2^viii^—Mo2—Mo3	117.29 (2)	Se4—Na2—Se4^x^	81.61 (16)
Se3—Mo2—Mo3	56.13 (2)	Se3^ix^—Na2—Se2^xviii^	111.08 (14)
Se1—Mo2—Mo3	132.31 (2)	Se4—Na2—Se2^xviii^	156.4 (2)
Mo3^viii^—Mo2—Mo3	58.76 (2)	Se4^x^—Na2—Se2^xviii^	84.93 (4)
Se5—Mo2—Mo1^iv^	91.085 (16)	Se3^ix^—Na2—Se2^xvi^	111.08 (14)
Se2—Mo2—Mo1^iv^	132.93 (2)	Se4—Na2—Se2^xvi^	84.93 (4)
Mo2^ix^—Mo2—Mo1^iv^	149.106 (14)	Se4^x^—Na2—Se2^xvi^	156.4 (2)
Mo2^viii^—Mo2—Mo1^iv^	100.94 (2)	Se2^xviii^—Na2—Se2^xvi^	100.51 (17)
Se2^viii^—Mo2—Mo1^iv^	49.978 (15)	Se3^ix^—Na2—Mo3^xvi^	146.9 (2)
Se3—Mo2—Mo1^iv^	93.55 (2)	Se4—Na2—Mo3^xvi^	116.51 (14)
Se1—Mo2—Mo1^iv^	47.329 (15)	Se4^x^—Na2—Mo3^xvi^	116.51 (14)
Mo3^viii^—Mo2—Mo1^iv^	101.18 (2)	Se2^xviii^—Na2—Mo3^xvi^	53.86 (9)
Mo3—Mo2—Mo1^iv^	149.02 (2)	Se2^xvi^—Na2—Mo3^xvi^	53.86 (9)
Se3—Mo3—Se3^ix^	176.87 (3)	Se3^ix^—Na2—Se3^xv^	162.9 (2)
Se3—Mo3—Se2	88.45 (2)	Se4—Na2—Se3^xv^	79.44 (13)
Se3^ix^—Mo3—Se2	93.31 (2)	Se4^x^—Na2—Se3^xv^	79.44 (13)
Se3—Mo3—Se2^x^	88.45 (2)	Se2^xviii^—Na2—Se3^xv^	79.14 (11)
Se3^ix^—Mo3—Se2^x^	93.31 (2)	Se2^xvi^—Na2—Se3^xv^	79.14 (11)
Se2—Mo3—Se2^x^	111.50 (3)	Mo3^xvi^—Na2—Se3^xv^	50.14 (8)
Se3—Mo3—Mo3^viii^	58.60 (4)	Se3^ix^—Na2—Na2^xvi^	116.92 (17)
Se3^ix^—Mo3—Mo3^viii^	118.27 (4)	Se4—Na2—Na2^xvi^	49.04 (6)
Se2—Mo3—Mo3^viii^	117.76 (2)	Se4^x^—Na2—Na2^xvi^	49.04 (6)
Se2^x^—Mo3—Mo3^viii^	117.76 (2)	Se2^xviii^—Na2—Na2^xvi^	107.97 (17)
Se3—Mo3—Mo3^ix^	118.60 (3)	Se2^xvi^—Na2—Na2^xvi^	107.97 (17)
Se3^ix^—Mo3—Mo3^ix^	58.27 (4)	Mo3^xvi^—Na2—Na2^xvi^	96.1 (2)
Se2—Mo3—Mo3^ix^	120.43 (2)	Se3^xv^—Na2—Na2^xvi^	45.99 (16)
Se2^x^—Mo3—Mo3^ix^	120.43 (2)	Se3^ix^—Na2—Na2^xii^	56.92 (17)
Mo3^viii^—Mo3—Mo3^ix^	60.0	Se4—Na2—Na2^xii^	49.04 (6)
Se3—Mo3—Mo2^xi^	118.045 (18)	Se4^x^—Na2—Na2^xii^	49.04 (6)
Se3^ix^—Mo3—Mo2^xi^	60.968 (17)	Se2^xviii^—Na2—Na2^xii^	129.74 (9)
Se2—Mo3—Mo2^xi^	149.86 (3)	Se2^xvi^—Na2—Na2^xii^	129.74 (9)
Se2^x^—Mo3—Mo2^xi^	59.121 (17)	Mo3^xvi^—Na2—Na2^xii^	156.1 (2)
Mo3^viii^—Mo3—Mo2^xi^	89.853 (16)	Se3^xv^—Na2—Na2^xii^	105.99 (16)
Mo3^ix^—Mo3—Mo2^xi^	61.335 (19)	Na2^xvi^—Na2—Na2^xii^	60.000 (1)
Se3—Mo3—Mo2^ix^	118.045 (18)	Se3^ix^—Na2—Mo1^xi^	96.57 (7)
Se3^ix^—Mo3—Mo2^ix^	60.968 (17)	Se4—Na2—Mo1^xi^	121.60 (17)
Se2—Mo3—Mo2^ix^	59.121 (17)	Se4^x^—Na2—Mo1^xi^	40.767 (18)
Se2^x^—Mo3—Mo2^ix^	149.86 (3)	Se2^xviii^—Na2—Mo1^xi^	44.758 (19)
Mo3^viii^—Mo3—Mo2^ix^	89.853 (16)	Se2^xvi^—Na2—Mo1^xi^	142.89 (16)
Mo3^ix^—Mo3—Mo2^ix^	61.335 (19)	Mo3^xvi^—Na2—Mo1^xi^	89.52 (8)
Mo2^xi^—Mo3—Mo2^ix^	112.93 (3)	Se3^xv^—Na2—Mo1^xi^	80.99 (8)
Se3—Mo3—Mo2^x^	60.536 (17)	Na2^xvi^—Na2—Mo1^xi^	78.76 (9)
Se3^ix^—Mo3—Mo2^x^	118.389 (19)	Na2^xii^—Na2—Mo1^xi^	85.87 (7)
Se2—Mo3—Mo2^x^	145.85 (3)	Se3^ix^—Na2—Mo1^ix^	96.57 (7)
Se2^x^—Mo3—Mo2^x^	57.914 (17)	Se4—Na2—Mo1^ix^	40.767 (18)
Mo3^viii^—Mo3—Mo2^x^	59.903 (18)	Se4^x^—Na2—Mo1^ix^	121.60 (17)
Mo3^ix^—Mo3—Mo2^x^	89.039 (15)	Se2^xviii^—Na2—Mo1^ix^	142.89 (16)
Mo2^xi^—Mo3—Mo2^x^	57.509 (17)	Se2^xvi^—Na2—Mo1^ix^	44.758 (19)
Mo2^ix^—Mo3—Mo2^x^	146.42 (3)	Mo3^xvi^—Na2—Mo1^ix^	89.52 (8)
Se3—Mo3—Mo2	60.536 (17)	Se3^xv^—Na2—Mo1^ix^	80.99 (8)
Se3^ix^—Mo3—Mo2	118.388 (19)	Na2^xvi^—Na2—Mo1^ix^	78.76 (9)
Se2—Mo3—Mo2	57.913 (17)	Na2^xii^—Na2—Mo1^ix^	85.87 (7)
Se2^x^—Mo3—Mo2	145.85 (3)	Mo1^xi^—Na2—Mo1^ix^	157.26 (17)
Mo3^viii^—Mo3—Mo2	59.903 (18)		

Symmetry codes: (i) *x*+1, *y*, *z*; (ii) *x*−*y*, *x*−1, −*z*+1; (iii) −*y*+1, *x*−*y*−1, *z*; (iv) −*x*+*y*+2, −*x*+1, *z*; (v) *y*+1, −*x*+*y*+1, −*z*+1; (vi) −*x*+*y*+1, −*x*, *z*; (vii) −*x*+2, −*y*, −*z*+1; (viii) −*y*+1, *x*−*y*, *z*; (ix) −*x*+*y*+1, −*x*+1, *z*; (x) *x*, *y*, −*z*+3/2; (xi) −*x*+*y*+1, −*x*+1, −*z*+3/2; (xii) −*x*+*y*, −*x*, *z*; (xiii) −*x*+1, −*y*, −*z*+1; (xiv) −*y*, *x*−*y*−1, *z*; (xv) *x*−1, *y*, *z*; (xvi) −*y*, *x*−*y*, *z*; (xvii) *x*−*y*, *x*, −*z*+1; (xviii) −*y*, *x*−*y*, −*z*+3/2.
